# Seroprevalence of Transfusion Transmissible Infections and Associated Risk Factors in Hospitalized Patients before Transfusion in Jinling Hospital Nanjing University: A Three-Year Retrospective Study

**DOI:** 10.3390/pathogens11060710

**Published:** 2022-06-20

**Authors:** Wei Wang, Xiaojun Kong, Guangchao Zhao, Xuelian Huang, Jun Yuan, Na Li, Xiaonan Zhang, Kaiyun Luo, Jianfeng Luan, Xuzhou Fan

**Affiliations:** 1 Department of Blood Transfusion Medicine, School of Medicine, Jinling Hospital, Nanjing University, Nanjing 210016, China; wwnjmu@126.com (W.W.); ZGC15150568868@126.com (G.Z.); hxl123fight@163.com (X.H.); soda_y@163.com (J.Y.); lina_transfusion@126.com (N.L.); nangua0808zhang@163.com (X.Z.); luokaiyunmm@126.com (K.L.); 2 Department of Blood Transfusion, The Affiliated Jiangning Hospital of Nanjing Medical University, Nanjing 211199, China; kxiaojun1988@163.com

**Keywords:** blood transfusion, transfusion transmissible infections, positive rate, high-speed centrifugation, confirmatory assays

## Abstract

Transfusion-transmitted infections (TTIs), such as hepatitis B virus (HBV), hepatitis C virus (HCV), human immunodeficiency virus (HIV), and treponema pallidum (TP), must be detected before blood transfusion. However, few studies have been conducted on the prevalence and accuracy of positive results in hospitalized patients. The purpose of this study was to investigate the real seroprevalence of TTIs among patients before blood transfusion and analyze the characteristics of false-positive results in Jinling Hospital, Nanjing University, China. TTI results were collected from medical records and analyzed retrospectively. Additionally, we also used confirmatory assays to verify the accuracy of positive results. The overall prevalence of TTI was 8.96%, which was related to gender and age. The real positive rates were 86.67% (HBV), 35.09% (HCV), 20.75% (HIV), and 100% (TP). Our results also showed that high-speed centrifugation can reduce the false-positive rate of HBsAg. In summary, the results demonstrated that the positive rates of TTIs in hospitalized patients are higher than those in the general population. We also confirmed the existence of false-positive results in serological screening for TTIs. The method of processing specimens through high-speed centrifugation could reduce the false-positive results of detecting antigens effectively.

## 1. Introduction

Blood transfusion is a significant method in modern medical care. Blood components, such as red blood cells, plasma, and platelets, are widely used in clinical practice [1]. However, blood-borne infectious pathogens may increase the residual risk of transfusion-transmitted infections (TTIs), such as viruses, bacteria, and parasites. Several effective pathogen reduction techniques (PRT) have been developed to achieve a broad-spectrum virucidal effect, such as the use of ultraviolet light and chemicals to induce damage in nucleic acid-containing agents, and heat to destroy the structural stability of viral proteins [2,3,4]. Moreover, blood screening techniques, including enzyme-linked immunosorbent assay (EIA) and nucleic acid amplification technology (NAT), have also been used to block the transmission of TTIs [5]. Laws and regulations on blood transfusion clearly stipulate that TTIs, such as hepatitis B virus (HBV), hepatitis C virus (HCV), human immunodeficiency virus (HIV), and treponema pallidum (TP), must be tested before blood transfusion to ensure the safety of blood recipients in many countries [6]. At present, the investigation of the positive rate of TTI markers among voluntary blood donors is attracting an increasing amount of attention [7]. However, in terms of patients receiving blood transfusion, who are most likely to be infected with blood-borne diseases, few studies have been conducted on the infection rate and the accuracy of positive results in this group [8]. The accuracy of the examination results before blood transfusion can not only prevent the superinfection of infectious diseases but also avoid medical accidents and disputes after blood transfusion.

Enzyme-linked immunosorbent assay (EIA) is one of the most commonly used serological methods to detect hepatitis B surface antigen (HBsAg), HCV antibody (anti-HCV), HIV antibody (anti-HIV), and syphilis antibody (anti-TP) in the laboratory [9]. In the process of EIA, there are various influencing factors, and it is easily affected by interfering substances, which may lead to errors in the results [10]. The physiological and pathological statuses of patients before blood transfusion are complicated, and the body conditions of different groups vary greatly, so the accuracy of positive TTI results has yet to be verified. Taking into account the safety of blood transfusion treatment, we carried out this retrospective study to determine the trend of positive rates of TTIs in the patient group and the accuracy of TTI test results. Therefore, we analyzed the results of HBsAg, anti-HCV, anti-HIV, and anti-TP in patients before possible blood transfusions in China during the period 2017–2019, aiming to improve the accuracy of the results of TTIs and support the validity of donor serological screening, so as to ensure the safety of blood transfusion. Besides, our study also provides epidemiological information on the hospitalized patient population compared to the general and blood donor populations.

## 2. Results

### 2.1. Patients’ Demographic Characteristics

A total of 197,372 patients received pre-transfusion screening for the TTIs (HBV, HCV, HIV, TP), of whom 115,951 (58.75%) were males and 81,421 (41.25%) were females. The age range of the screened subjects is from 0 to 99 years old, the majority of them were 45 years old and above and accounted for over 60% of all the subjects. A total of 55,256 patients received blood transfusions (Table 1).

### 2.2. Positive Rates and Trends of TTIs

A total of 197,372 cases of patients, HBsAg, anti-HCV, anti-HIV, and anti-TP, were 17,676 cases of positive test results. The positive rate with respect to the total TTIs was 8.96%, the positive rates of HBsAg, anti-HCV, anti-HIV, and anti-TP were 5.36, 0.92, 0.05, and 2.62%, respectively. From 2017 to 2019, the positive rate of HBsAg decreased from 5.57% to 5.20% (*p* < 0.05), and the positive rate of anti-HCV decreased from 1.07% to 0.82% (*p* < 0.001). On the contrary, the positive rate of anti-TP increased from 2.25% to 2.86% (*p* < 0.001). The positive rate of anti-HIV remained relatively steady over the 3-year study period (*p* = 0.41) (Table 2).

There were significant differences in the positive rates of TTIs in different age groups (*p* < 0.001). Among them, the positive rate of anti-HCV was the highest in the group from 46 to 60 years old, the positive rate of HBsAg was the highest in the group from 36 to 45 years old, and the highest positive rate of anti-HIV was found in the group from 26 to 35 years old. When the age is greater than 60 years old, the anti-TP positive rate became the highest group (Table 2).

**Figure 1:**
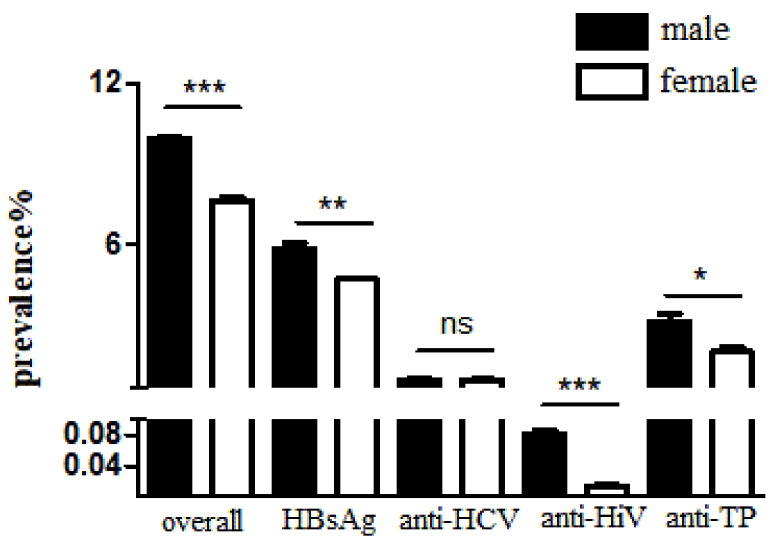
The positive rates of HBsAg, anti-HCV, anti-HIV, anti-TP in different genders. Note: hepatitis B surface antigen, HBsAg; HCV antibody, anti-HCV; HIV antibody, anti-HIV; syphilis antibody, anti-TP. Statistical significance was expressed as follows: * *p* < 0.05, ** *p* < 0.01, *** *p* < 0.001, ns *p* > 0.05.

The overall positive rates of TTIs for males were 9.92% and for females were 7.58%. The positive rates of HBsAg, anti-HIV, and anti-TP in the males were significantly higher than those in the females (*p* < 0.05) (Figure 1).

### 2.3. Positive Rates of TTIs in Different Departments

The total positive rate of TTIs in nephrology was the highest at 9.91%, and the lowest positive rate in pediatric infections was 0.59%. There was a significant difference in the positive rates of TTIs (HBsAg, anti-HCV, anti-HIV, and anti-TP) among different departments (*p* < 0.001). The anti-HCV positive rate (1.84%) in the nephrology department was the highest, which was more than twice that of other departments. The anti-TP positive of internal medicine (2.85%) was higher than most departments, and general surgery had a higher HBsAg positive rate (6.64%) than all other departments (Table 3).

### 2.4. The Results of the Confirmatory Assays

Sixty cases of HBsAg, 57 cases of anti-HCV, 53 cases of anti-HIV, and 54 cases of anti-TP were tested by the confirmatory assays to verify the accuracy of positive results. The positive rates of TTIs (HBsAg, anti-HCV, anti-HIV, and anti-TP) confirmation tests were 86.67%, 35.09%, 20.75%, and 100%, respectively. The accuracy of the positive results of anti-HIV and anti-HCV with S/CO values between 1.0 and 7.0 was significantly lower than that of specimens with S/CO values greater than 7.0 (*p* < 0.05). When the S/CO value was higher than 5.0, the positive accuracy rate of HBsAg was 91.67%, and the positive accuracy rate of anti-TP was 100%, which was much higher than the anti-HCV and anti-HIV positive accuracy rates (Table 4).

### 2.5. The Results of High-Speed Centrifugation

**Figure 2:**
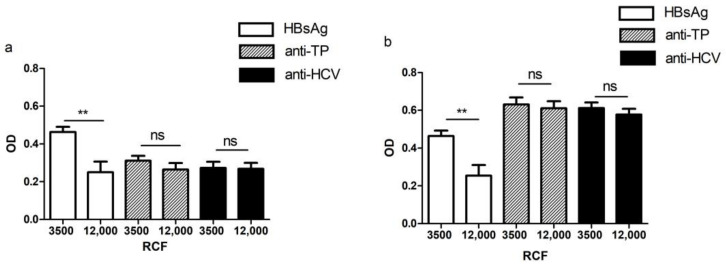
The effect of high-speed centrifugation on the OD value of TTIs. (**a**) The OD value of TTIs ranges 1.0 from 3.0; (**b**) the OD value of TTIs ranges from 3.0 to 5.0. Note: transfusion transmissible infections, TTIs; optical density, OD. Statistical significance was expressed as follows: ** *p* < 0.01, ns *p* > 0.05.

Forty-one samples with S/CO values in the range of 1.0–5.0 were centrifuged at 12,000 rpm and re-examined by EIA. The OD value of HBsAg after high-speed centrifugation with an S/CO value in the range of 1.0–5.0 was significantly lower than that of low-speed centrifugation (*p* < 0.05) (Figure 2). Moreover, the positive rate of HBsAg after high-speed centrifugation was 69.23%, which was significantly lower than that of low-speed centrifugation (*p* < 0.05) (Table 5). However, the OD values and positive rates of anti-HCV and anti-TP after high-speed centrifugation had no differences (*p* > 0.05), so high-speed centrifugation has no effect on the re-examination of these items.

## 3. Discussion

TTIs, one of the severe adverse reactions of blood transfusion, pose a constant threat to the safety of blood transfusion [11]. Although blood products undergo a strict screening process, there is still an existential risk of spreading TTIs [12]. The results of TTIs before blood transfusion constitute important evidence to reduce or avoid medical disputes caused by blood transfusion infections. Therefore, this study takes the TTI infection data of patients who are less studied presently as the research object. We found that the TTI infection rate of patients was much higher than that of the voluntary blood donors, and there were great differences between age, gender, and disease. We further verified the positive results of infection and found that they had a certain false-positive rate. The method of processing specimens using high-speed centrifugation can effectively reduce the false-positive results of detecting antigens.

From 2013 to 2017, the prevalence of HBV infection in the general population in China was 6.89% (95% CI:5.84–7.95%) [13]. In our study, the positive rate of HBsAg in the patients was 5.36%, which is roughly the same as the positive rate in the general population. However, it was much higher than the rate of voluntary blood donors, which was 0.51% [14]. This study showed that the positive rate of anti-HCV was 0.92%, which is slightly higher than the 0.61% reported in the general population of China in 2009 [15]. Recent studies have shown that there are approximately 185 million infections worldwide, and the HCV seroprevalence rate has risen to 2.8% in the past decade [16]. Our data further support that China is a low-endemic area of HCV. However, the number of HCV infections is still at a very high level due to the number of people in China. The total positive rates of anti-HIV and anti-TP were 0.05% and 2.62%, respectively. The Joint United Nations Programme on HIV/AIDS (UNAIDS) reports that 75.7 million people are infected with HIV, an infection rate of nearly 1% [17]. HIV has become a generalized epidemic in the general population (usually <1% prevalence) in China [18]. According to large-scale data, the global average incidence of syphilis is approximately 1.11% [19], which is lower than the positive rate of our patients. All the epidemiological data in this study will provide a reference for the study of the prevention, control, and treatment of TTIs.

Our research shows that gender, age, and disease status all have an impact on the positive rate of TTIs. The overall infection rate of TTIs in men (9.92%) is significantly higher than that in women (7.58%). Especially in the two sexually transmitted diseases (HIV and syphilis), the HIV infection rate of men (0.08%) is much higher than that of women (0.01%), and the anti-TP infection rate of men (3.08%) is also higher than that of women (1.97%), which may be influenced by the prevalence of men who have sex with men (MSM) [20]. It is worth noting that age also has a certain impact on the infection of TTIs. In this study, the positive rate of HBsAg reached the highest level in 46–60-year-olds. The national vaccination program implemented in 1992 by China caused a significant decrease in the infection rates of the young group. At present, Chinese neonates are being vaccinated at the birth dose and in a three-dose schedule to achieve the goal of elimination [21]. Our data confirmed that this is an effective measure to prevent the spread of HBV. In addition, we are concerned that the positive rate of anti-TP is positively correlated with age, and the positive rate of anti-TP in patients over 60 years old is significantly higher than that of other age groups. According to reports, common medical diseases in the elderly population can cause biological false positives for syphilis, such as systemic lupus erythematosus (SLE), cancers, malaria, other treponemal infections, HIV, and HCV [22]. These data suggest that the accuracy of the positive results of anti-TP in elderly patients warrants further verification.

Differences in the physiological and pathological status of patients will have a certain impact on the accuracy of TTI detection; for example, whether the patient has received blood transfusions, has been infected with TTIs, and has taken immuno-suppressive drugs. Studies have shown that RBC products contain a multitude of immunomodulatory mediators that interact with and alter immune cell function [23]. A study also showed that women with HCV are more likely to have a biological false-positive syphilis test than women without HCV [24]. Similarly, some immuno-suppressive drugs can cause false positives of TTIs [25]. We suspect that this is because the body’s immune substances interfere with the serum screening of TTIs. In our results, the positive rates of TTIs in the nephrology department were significantly higher than those of other departments. The damage to the body’s immune system and bacterial infections often lead to kidney disease [26]. Kidney diseases are often accompanied by disorders of the immune system of the organism, and the production of special antibodies may interfere with the normal response of EIA, which will result in false-positive results [27]. This suggests that the disease condition of patients may have an impact on the accuracy of the TTI detection rate. For departments with abnormal TTI rates, the accuracy of the results warrants further verification to avoid misjudgments caused by the pathological state of patients interfering with the results.

In view of the above factors, which may be related to the positive result, we selected some specimens for confirmation experiments to verify the accuracy of the positive result. The study found that the accuracy of the positive HBsAg result reached 86.67%. Among them, the false-positive rate of HBsAg and S/CO of less than 7.0 in the EIA method was 8.33%, which meant that there were false-positive results at low concentrations. The laboratory should formulate a reasonable S/CO value to determine the result according to the specific situation, and the sample with a lower detection value should be re-examined using a confirmatory experiment to comprehensively determine the experimental result. However, the positive rates of anti-HCV and anti-HIV by confirmation tests were 35.09% and 20.79%, respectively. Among them, the false-positive rates of specimens with detection values in the range of S/CO of less than 7.0 were as high as 87.5% (anti-HCV) and 100% (anti-HIV). Therefore, the positive results of EIA alone should not be used to determine whether a patient is infected. A more specific confirmatory test is needed to verify the accuracy, so as to avoid possible medical disputes. The false-positive rates of anti-TP were 26.67% (1.0 < S/CO ≦ 3.0) and 8.33% (3.0 < S/CO ≦ 5.0), which were the lowest among the TTIs. We recommend that in specimens with S/CO values of less than 5.0, the reverse screening strategy of syphilis, that is, EIA or chemiluminescence, should be used as the preliminary screening test for anti-TP. If the result is positive, then nonspecific tests, such as RPR or TRUST, should be used for syphilis detection. If it is negative, then a more specific anti-TP detection method, such as TPPA, should be used for confirmation to reduce the false-positive results of anti-TP.

As a routine primary screening method for TTIs, EIA was widely used in clinical testing and blood station institutions [28]. However, EIA had high sensitivity and many interference factors, which cause false-positive reactions, such as sample addition, reaction temperature, reaction time, thoroughness of washing, hemolysis of specimens, and reagent factors. Therefore, many countries choose to use NAT with a higher specificity and shorter window period in blood donor screening [5]. It is also appropriate to suggest that the serological test with high sensitivity should then be confirmed through highly specific confirmatory tests in order to limit the number of false positives. However, the confirmation tests were not carried out in most laboratories due to the influence of experimental conditions and the skills of experimenters. Therefore, we proposed a method to solve or reduce false-positive results based on the original laboratory technical conditions, that is, the method of using high-speed centrifugation. Studies had shown that high-speed centrifugation at 10,000 rpm for 10 min can minimize the influence of fibrin, blood cells and their fragments, and other particles that may exist in the blood, on the detection of antigens and antibodies [29]. Therefore, in this study, we centrifuged the specimens at 12,000 rpm for 10 min and re-examined them using the original method. The results showed that the detection value of HBsAg decreased significantly, and the rate positive of HBsAg was also reduced significantly, suggesting that high-speed centrifugation could effectively avoid the influence of interfering substances on HBsAg detection. We also noticed that high-speed centrifugation had no obvious effect on the detection of antibodies, such as anti-HCV and anti-TP. This is because the molecular weight of antibodies is much smaller than that of antigens. It may be difficult to avoid interferences through centrifugation alone. Further research is required to solve such problems. 

However, the empirical results reported herein should be considered in light of some limitations. In fact, the confirmatory are not regular testing projects in our hospital, the cost of the experiment and the availability of valid samples were considered, and only 224 specimens were selected for confirmatory tests finally. In addition, we have not set up strict TTIs inclusion criteria, all TTIs detection data were collected from medical records, so the TTIs infection status of some people in our cases may be unknown. These limitations will be further improved in future studies.

## 4. Materials and Methods

### 4.1. Sample and Data Collection

The study did not set strict TTIs inclusion criteria. We collected a total of 197,372 cases of TTI detection data from medical records in Jinling Hospital who planned to receive blood transfusions, surgery, or other treatments from 2017 to 2019. The status of TTIs infection before hospitalization in some patients is unknown. All serum specimens were separated through centrifugation at 3000 revolutions per minute (rpm) for 5 min.

### 4.2. Serologic Assays

Serum specimens were assessed for HBsAg, and antibodies to HCV (anti-HCV), HIV types 1 and 2 (anti-HIV1/2), and anti-TP by enzyme-linked immunosorbent assay (EIA) on the FAME24/20 (Hamilton, Switzerland). All the reagents for the project were provided by InTec Inc Company and approved by the State Food and Drug Administration of China. All experimental steps were carried out according to the manufacturers’ information. All the results of the TTI markers were expressed as the S/CO ratio. When S/CO < 1, it indicated a nonreactive result and vice versa. All reagents were from quality batches and were used within the validity period. Similarly, the positive, negative, and blank controls were signed. The sample was tested using double-well potential, and the results all reached S/CO ≥ 1 and were judged as positive.

### 4.3. Confirmatory Assays

HBsAg was confirmed through the neutralization confirmation test (Lizhu, Zhuhai, China), which used the principle of specific antibody neutralization to confirm the presence of HBsAg. The neutralization confirmation test is based on EIA, but is more specific, and is used to confirm the accuracy of weak reaction results [30]. As there are excessive antibodies of HBV in the reagent, it can neutralize the HBsAg in the specimen, so the OD value of the test will be reduced. The experimental results are related to the inhibition rate. If the inhibition rate is greater than 50%, it is a positive result, but if the inhibition rate is less than 50% and the optical density (OD) value is less than 2.0, it is a negative result. The reactive anti-HCV results were confirmed through line immunoassay (LIA, Wantai, Beijing, China), which has high sensitivity and specificity [31]. The LIA is an alternate to Western blot (WB), but this technique utilizes purified antigens as well as synthetic peptides and is coated onto a nylon strip with plastic backing in discrete lines. The result is negative if there is no reactive protein band, and it is positive with at least two strongly reactive protein bands. The weak reactivity of only one protein band or two and/or more protein bands is considered uncertain. If the anti-HIV result was positive, we retested the serum sample to confirm whether there was HIV infection through Western blot (WB) analysis, which was carried out in the Laboratory of Nanjing Center for Disease Control and Prevention (CDC). We also used LIA (Eueoimmun, Lübeck, Germany) to detect anti-TP in the EIA-positive patients for further verification. The interpretation of the results is similar to that of anti-HCV, which is determined by the appearance of protein strips.

### 4.4. High-Speed Centrifugation

In order to avoid the influence of fibrin, blood cells, cell debris, and other particles, which may be present in the blood on the detection of antigens and antibodies, we performed high-speed centrifugation on 13 samples of HBV, 16 samples of HCV, and 12 samples of TP (1 < S/CO ≦ 5) at 12,000 rpm for 10 min for detection (Centrifuge 5415R, Eppendorf, Germany). Then, we checked the marker reaction again using EIA.

### 4.5. Statistical Analysis

All statistical analyses were generated using GraphPad Prism software 5.0 (GraphPad Software, San Diego, CA, USA). The percentage of TTIs was determined through frequency distributions. The correlation between the positive rate of TTIs, clinical status, and laboratory variables was analyzed using the chi-square test. The means and standard errors of means (SEMs) were compared with a Student’s *t*-test between the two groups. Variables with a *p*-value of less than 0.05 were considered statistically significant.

## 5. Conclusions

We reported the infection status of patients with TTIs in eastern China. Our results suggested that there was a false-positive rate for positive results in different populations. Age, gender, and disease status all had an impact on the positive results. We recommend that the laboratory establish a control relationship between the test value and the actual result. Moreover, the S/CO value should be set reasonably according to the actual situation to avoid false-positive results as much as possible. In addition, we propose that the high-speed centrifugation method could be used to process specimens, which provides a certain reference for reducing false-positive HBsAg results. In summary, when studying the distribution of TTI infection in a certain area, the false-positive rates should be considered comprehensively, so as to obtain more accurate epidemiological information.

## Figures and Tables

**Table 1:** Distribution of TTIs by gender and age groups in Jinling Hospital, 2017–2019.

Characteristic	No.	%
*Gender*		
Male	115,951	58.75
Female	81,421	41.25
*Age*		
0–5	303	0.15
6–12	1688	0.86
13–18	2337	1.18
18–25	15,426	7.82
26–35	23,250	11.78
36–45	26,387	13.37
46–60	60,057	30.43
>60	67,924	34.41
*Total*	197,372	100
*Transfusion*	55,256	27.99
Red blood cells	27,613	13.99
Plasma	21,607	10.95
Platelets	6036	3.06

**Table 2:** Positive rates of HBsAg, anti-HCV, anti-HIV, and anti-TP among patients.

Year	Number of Patients Tested	Total Number of Positives (%)	HBsAg * (%)	Anti-HCV * (%)	Anti-HIV * (%)	Anti-TP * (%)
2017	63,421	5669 (8.94)	3533 (5.57)	680 (1.07)	31 (0.05)	1425 (2.25)
2018	66,877	6020 (8.75)	3561 (5.32)	581 (0.87)	42 (0.06)	1836 (2.75)
2019	67,074	5987 (8.93)	3486 (5.20)	551 (0.82)	32 (0.05)	1918 (2.86)
Total	197,372	17,676 (8.96)	10,580 (5.36)	1812 (0.92)	105 (0.05)	5179 (2.62)
*χ^2^*		0.27	9.22	25.23	1.763	53.71
*p*		>0.05	<0.05	<0.05	>0.05	<0.05
Male						
2017	36,936	3623 (9.81)	2271 (6.15)	405 (1.10)	29 (0.08)	918 (2.49)
2018	39,163	3867 (9.87)	2289 (5.84)	346 (0.88)	37 (0.09)	1195 (3.05)
2019	39,852	4015(10.1)	2204 (5.53)	325 (0.82)	28 (0.07)	1458 (3.66)
Total	115,951	11,505 (9.92)	6764 (5.83)	1076 (0.93)	94 (0.08)	3571 (3.08)
*χ^2^*		1.67	13.34	17.73	1.47	88.55
*p*		>0.05	<0.05	<0.05	>0.05	<0.05
Female						
2017	26,485	2038 (7.69)	1262 (4.76)	267 (1.01)	2 (0.01)	507 (1.91)
2018	27,714	2153 (7.77)	1272 (4.59)	235 (0.85)	5 (0.02)	641 (2.31)
2019	27,222	1980 (7.27)	1282 (4.71)	234 (0.86)	4 (0.01)	460 (1.69)
Total	81,421	6171 (7.58)	3816 (4.69)	736 (0.90)	11 (0.01)	1608 (1.97)
*χ^2^*		5.56	0.98	4.78	1.15	28.29
*p*		>0.05	>0.05	>0.05	>0.05	<0.05
Age						
0–5	303	0	0 (0)	0 (0)	0 (0)	0 (0)
6–12	1688	12 (0.71)	6 (0.36)	1 (0.06)	0 (0)	5 (0.30)
13–18	2337	27 (1.16)	19 (0.81)	6 (0.26)	0 (0)	2 (0.09)
18–25	15,426	405 (2.63)	263 (1.70)	57 (0.37)	10 (0.06)	75 (0.49)
26–35	23,250	1472 (6.33)	1091 (4.69)	108 (0.46)	25 (0.11)	248 (1.07)
36–45	26,387	2815 (10.67)	1938 (7.34)	261 (0.99)	26 (0.10)	590 (2.24)
46–60	60,057	6740 (10.89)	4214 (7.02)	712 (1.19)	27 (0.04)	1787 (2.98)
>60	67,924	6205 (9.14)	3049 (4.49)	667 (0.98)	17 (0.03)	2472 (3.64)
*χ^2^*		1743.6	1235.6	180.0	36.71	908.7
*p*		<0.05	<0.05	<0.05	<0.05	<0.05

* Hepatitis B surface antigen, HBsAg; HCV antibody, anti-HCV; HIV antibody, anti-HIV; syphilis antibody, anti-TP.

**Table 3:** Positive rates of TTIs in different departments.

Department	Number of Patients Tested	Total Number of Positives (%)	HBsAg * (%)	Anti-HCV * (%)	Anti-HIV * (%)	Anti-TP * (%)
Nephrology	26,327	2609 (9.91)	1587 (6.03)	485 (1.84)	15 (0.06)	522 (1.98)
General surgery	19,316	1880 (9.73)	1283 (6.64)	160 (0.83)	4 (0.02)	433 (2.24)
Orthopedics	15,625	1294 (8.28)	833 (5.33)	103 (0.66)	7 (0.04)	351 (2.25)
Obstetrics and gynecology	5415	380 (7.02)	242 (4.47)	22 (0.41)	0 (0)	116 (2.14)
Pediatrics	1533	9 (0.59)	3 (0.20)	3 (0.20)	0 (0)	3 (0.20)
Internal medicine	46,390	4165 (8.98)	2430 (5.24)	388 (0.84)	25 (0.05)	1322 (2.85)
Surgery	29,135	2479 (8.51)	1608 (5.52)	227 (0.78)	5 (0.02)	639 (2.19)
Others	53,631	4860 (8.33)	2594 (4.83)	424 (0.79)	49 (0.09)	1793 (3.34)
*χ^2^*		217.1	206.7	304.1	29.62	241.5
*p*		<0.05	<0.05	<0.05	<0.05	<0.05

* Hepatitis B surface antigen, HBsAg; HCV antibody, anti-HCV; HIV antibody, anti-HIV; syphilis antibody, anti-TP.

**Table 4:** Positive rates of the confirmatory assays.

S/CO	HBsAg * (%)		Anti-HCV * (%)		Anti-HIV * (%)		Anti-TP * (%)	
	EIA *	Neutralization Tested	EIA	LIA*	EIA	WB *	EIA	LIA
1.0–3.0	12	7 (58.33)	15	0 (0.00)	14	0 (0.00)	15	11 (73.33)
3.0–5.0	15	13 (86.67)	12	0 (0.00)	9	0 (0.00)	12	11 (91.67)
5.0–7.0	12	11 (91.67)	8	1 (12.5)	9	0 (0.00)	9	9 (100)
>7.0	21	21 (100)	22	19 (86.36)	15	11 (73.33)	18	18 (100)
Total	60	52 (86.67)	57	20 (35.09)	53	11 (20.75)	54	54 (100)
*χ^2^*		11.83		41.78		30.64		7.630
*p*		<0.05		<0.05		<0.05		>0.05

* Hepatitis B surface antigen, HBsAg; HCV antibody, anti-HCV; HIV antibody, anti-HIV; syphilis antibody, anti-TP; enzyme-linked immunosorbent assay, EIA; line immunoassay, LIA; Western blot, WB.

**Table 5:** Positive rates of the high-speed centrifugation.

Project	Number	S/CO	Number of Positives in Low-Speed Centrifugation	Positive Rate (%)	Number of Positives in High-Speed Centrifugation	Positive Rate (%)	Statistical Result
HBsAg *	13	1.0–5.0	13	100	9	69.23	*χ^2^* = 4.727
							*p* < 0.05
anti-HCV *	16	1.0–5.0	16	100	15	93.75	*χ^2^* = 1.032
							*p* < 0.05
anti-TP *	12	1.0–5.0	12	100	12	100	/

* Hepatitis B surface antigen, HBsAg; HCV antibody, anti-HCV; syphilis antibody, anti-TP.

## Data Availability

Not applicable.
